# Eupatorium Perfoliatum

**Published:** 1887-02

**Authors:** J. T. Kent


### EUPATORIUM PERFOLIATUM.

A Lecture by Professor J. T. Kent, A. M., M. D.

[Reported by C. S. Durand.]

This remedy is good for chills. So is Quinine, they say. I have my doubts about it.

This remedy conforms to a low state of the body coming on, not with great rapidity, but rather slowly, such as you will find in marshy localities. It conforms to the marsh miasm, producing forms of chills and fever. It produces a very severe form of ague, and you will need to be conversant with this remedy, because when it appears no other remedy will cure your case.

It does not produce the typical chill, fever, and sweat of Quinine—only as an exception. A profuse sweat following the chill and fever is the exception in Eupat. Scanty sweat is characteristic; but it is still more characteristic for it to produce a chill and fever with scarcely any sweat. When there has been a mild chill, there may be a good, profuse sweat; but when it appears in the aggravated form, like you have it in the marshy districts, in the low lands of the Western States, river bottoms, etc., you will have a violent chill and scarcely any sweat.

One of the grandest features running through this remedy is aching in the bones as if they would break, and hence it conforms to what the people commonly called the "break-bone" fever; and the common people have used it since early times for "break-bone" fever, and they have hence called it "bone-set."

Nearly always it is associated with disorders of the stomach, with a gastric headache. It produces a gastro-duodenal catarrh, hepatic congestion; hence, great yellowness of the skin and sclerotica; in fact, the eyes become so yellow that you notice it immediately upon the patient's entering your office. In an old case associated with this there is terrible aching and soreness in the eyes. Now this yellowness of the skin is associated with a stomach disorder, wherein, if he eat until his stomach is filled, he will suffer from terrible pains—aching in the stomach, which is relieved by vomiting of food, and then bile.

Vomiting bile is characteristic, and is a part of the red string of this remedy; in fact, he vomits enormous quantities of bile. Drinking of cold water is followed by severe pains in the stomach, nausea, and great vomiting; vomiting of water drank, mixed with bile. Thirst for cold drinks is a prominent feature, but for warm drinks it occurs only as an exception. But there is sometimes thirst for warm drinks, which relieve. Thirst for cold water during all stages of intermittent is not uncommon, and has been clinically verified. The characteristic, however, is thirst before the chill and during the chill and heat.

Another prominent characteristic in relation to this is thirst for large draughts of cold water before and during the chill, which is vomited at the close of the chill, or as the fever is about to come on. Of course, he has drank water and been drinking water till his stomach is full, which has not quenched his thirst, and he continues to drink until about the close of the chill, when there is a relaxation. A reaction begins to occur, and he vomits immense quantities of water and bile. There is a bitter taste in the mouth at all times. The vomit at the close of the chill commonly relieves him, so that he will go on through the fever and sweat, if sweat be present, without much vomiting. Again, it has an irritable stomach that continues through the heat and until the apyrexia.

Another grand feature relating to this drug is its violent headache, which comes on before the chill. This continues through all the stages, but is not so prominent during the chill and heat as during the sweat; hence, it is said that the sweat aggravates the headache. The sweat, when it is present, relieves all the other symptoms, the bone-pains, the nausea, anxiety, and thirst, as a rule, but not the headache.

In Natrum mur. the sweat relieves all the symptoms. But in Eupat. the sweat relieves all the symptoms except the headache, which is made worse. There you have two very distinct features. These headaches are not always present in Natrum mur., nor in every case of Eupat., but when all the symptoms are relieved except the headache, and that is made worse by the sweat, that is not a Natrum mur. case.

There is another feature as to the time for which this remedy is to be selected. In Eupat. he may have a chill almost any time, more commonly in the forenoon; but it has an evening chill, and there is a peculiar chill that is characteristic as to time, and that is an alternation of time. There will be a chill from seven to nine one morning, very violent, and the next day at twelve a light chill with not so many of the marked features. You will find no other remedy like this. I have seen that several times in intermittent fever, and Eupat. has always cured it. When you see this peculiarity as to time you will be almost certain to see the other characteristics of Eupat.

Now there is another peculiarity that marks the general state. There is great restlessness without relief from motion. You may have a fever with this restlessness so marked that you will be led to think of Rhus or Arsenic. But this condition does not depend so much upon innate restlessness, like Rhus and Arsenic, as it does upon the condition of the bones; his bones ache so—the pain is indescribable—they feel as if they would break, and he moves and moves, and moves continually—keeps in motion without relief.

Rhus has bone pains, though not in so marked a degree as Eupat.; but they are relieved by motion. Now you will be apt to associate this remedy (Eupat.) with Arnica, with Rhus, and Arsen.

The Eupat. patient moves because he can't keep still—his bones ache so. Although he gets no relief by moving, he seems to bear his pains more easily in motion than when at rest.

In Arnica the patient moves because of the soreness of the body. If you ask the Arnica patient why he moves, he will tell you he has been lying so long in one place that he is sore—that his flesh is sore. This soreness becomes so great that he turns over and seeks a new place. He gets relief for a moment, but by and by the soreness begins again, and he must hunt a new place. Although he is lying on feathers, or a new mattress, he can't keep still on account of the soreness.

Arsenic has innate restlessness. He is compelled to move because he can't keep still. But there is no aching of the bones with the restlessness; it is associated with an anguish of the flesh and mind—all over him; he is full of anguish, of anxiety and restlessness; he can't keep still.

Now, Eupat. is not a big remedy; it has not an extensive history, but it will conform to some of our low types of intermittent fever.

It has some chest symptoms that may be brought out with many of its complaints. It has a rough, scraping cough, chest sore—must support it with the hands. This is like Bryonia—must support the chest with the hands. This has been verified many times. Then it has heat about the soles of the feet and top of the head, like Sulphur.

Another feature that is sometimes quite characteristic in relation to the chill is, that the chill spreads from the back.

If you have a hectic fever with night-sweats from a suppressed ague, an ague that has been suppressed by Quinine, and night-sweats come on, with bone pains and aching all over, yellowness of the skin, and the general bilious state that has been described to you—gastric and hepatic symptoms—then you will think of Eupatorium.

There is a symptom that might make you think of Belladonna. The pains come suddenly and go suddenly. It has sharp, cutting pains in its pathogenesis. Bell. has this as a characteristic. Chamomilla has it also in a slight degree.

I have only given you a little of this remedy. But I believe all of you will be able to see its peculiar and distinguishing features. You will see that it does not produce a Quinine chill; that it does not produce an Arnica chill; that it does not produce an Ipecac. chill; but that it produces its own kind of chill with its own peculiarities.

